# Investigating oxygen reduction pathways on pristine SOFC cathode surfaces by *in situ* PLD impedance spectroscopy†

**DOI:** 10.1039/d1ta07128a

**Published:** 2021-11-05

**Authors:** Matthäus Siebenhofer, Christoph Riedl, Alexander Schmid, Andreas Limbeck, Alexander Karl Opitz, Jürgen Fleig, Markus Kubicek

**Affiliations:** Institute of Chemical Technologies and Analytics, TU Wien Vienna Austria matthaeus.siebenhofer@tuwien.ac.at markus.kubicek@tuwien.ac.at; CEST Centre of Electrochemistry and Surface Technology Wr. Neustadt Austria

## Abstract

The oxygen exchange reaction mechanism on truly pristine surfaces of SOFC cathode materials (La_0.6_Sr_0.4_CoO_3−*δ*_ = LSC, La_0.6_Sr_0.4_FeO_3−*δ*_ = LSF, (La_0.6_Sr_0.4_)_0.98_Pt_0.02_FeO_3−*δ*_ = Pt:LSF, SrTi_0.3_Fe_0.7_O_3−*δ*_ = STF, Pr_0.1_Ce_0.9_O_2−*δ*_ = PCO and La_0.6_Sr_0.4_MnO_3−*δ*_ = LSM) was investigated employing *in situ* impedance spectroscopy during pulsed laser deposition (i-PLD) over a wide temperature and *p*(O_2_) range. Besides demonstrating the often astonishing catalytic capabilities of the materials, it is possible to discuss the oxygen exchange reaction mechanism based on experiments on clean surfaces unaltered by external degradation processes. All investigated materials with at least moderate ionic conductivity (*i.e.* all except LSM) exhibit polarization resistances with very similar *p*(O_2_)- and *T*-dependences, mostly differing only in absolute value. In combination with non-equilibrium measurements under polarization and defect chemical model calculations, these results elucidate several aspects of the oxygen exchange reaction mechanism and refine the understanding of the role oxygen vacancies and electronic charge carriers play in the oxygen exchange reaction. It was found that a major part of the effective activation energy of the surface exchange reaction, which is observed during equilibrium measurements, originates from thermally activated charge carrier concentrations. Electrode polarization was therefore used to control defect concentrations and to extract concentration amended activation energies, which prove to be drastically different for oxygen incorporation and evolution (0.26 *vs.* 2.05 eV for LSF).

## Abstract 2

Temperature and *p*(O_2_) dependences of the oxygen exchange kinetics of SOFC cathode materials were correlated with point defect concentrations. Defect chemical contributions were identified as a major part of effectively measured activation energies.

## Graphical abstract

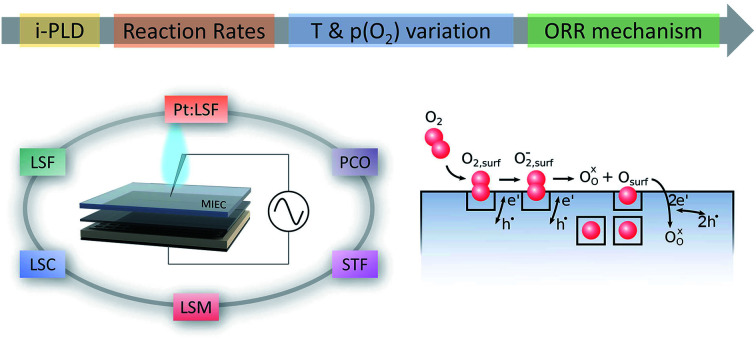

## 1. Introduction

The kinetics of the oxygen exchange reaction (OER) is crucial for a multitude of applications in energy- and environment-related technologies, *e.g.* electrode materials for solid oxide fuel cells (SOFCs),^1–3^ solid oxide electrolysis cells (SOECs)^4,5^ or oxygen permeation membranes.^6,7^ The overall reaction can be written as1
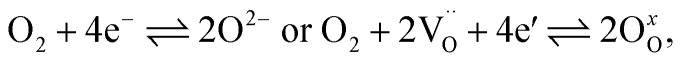
in common notation or in Kröger–Vink notation, respectively.^8^ The exact mechanism of the OER, however, is surprisingly complex and still not fully resolved (although the reaction itself seems quite simple). A comprehensive understanding of the processes occurring during these reactions (particularly of the rate determining steps) is thus essential with regard to the optimization of components for energy-related applications and to develop materials with satisfactory activity and stability. In the case of SOFCs, virtually all cathode materials are oxides and the predominant part of those possess perovskite structure.^3^ For many of those materials, the bottleneck in SOFC operation is the aforementioned surface reaction, where oxygen is incorporated into the electrode.^9^ From the nature of the reaction itself, it is evident that it must be comprised of several reaction steps: (a) diffusion of oxygen through the gas phase to the electrode surface, (b) adsorption of oxygen on the electrode surface, (c) dissociation of O_2,ads_, (d) charge transfer from the electrode to adsorbed oxygen and (e) incorporation into the lattice.^10–14^ However, the exact order and the detailed kinetics of all steps after (b) are difficult to unravel due to the complex interactions between electronic and ionic defects, especially with regard to the identification of the rate determining step. To further complicate the matter, a wide variety of cathode materials are used, and it is unknown if the oxygen surface exchange reaction proceeds similarly on all materials. Additionally, even for a given cathode composition, the surface structure and chemistry can vary substantially and multiple degradation but also activation phenomena may occur.

OER reaction rates can be measured by different experimental techniques such as tracer diffusion,^15–17^ relaxation experiments^18–20^ and impedance spectroscopy^12,21–24^ performed in different atmospheres and at different temperatures. All these measurement techniques yield different surface exchange coefficients (which are themselves often not straightforwardly interpretable^25^). They pose, in combination with the reaction order with regard to oxygen partial pressure, the basis for a reaction mechanism analysis. However, surfaces investigated in such experiments are usually far from undisturbed or pristine, for example due to degradation phenomena like Sr-segregation^26–31^ or due to various poisoning effects,^32–35^ potentially obscuring the true capabilities and properties of SOFC cathode materials.

In this work, we present the results of *in situ* impedance spectroscopic measurements on pristine, dense thin films immediately after deposition, *i.e.* still in the PLD chamber (i-PLD).^36–38^ The oxygen exchange kinetics of La_0.6_Sr_0.4_CoO_3−*δ*_ (LSC), La_0.6_Sr_0.4_FeO_3−*δ*_ (LSF), (La_0.6_Sr_0.4_)_0.98_Pt_0.02_FeO_3−*δ*_ (Pt:LSF), SrTi_0.3_Fe_0.7_O_3−*δ*_ (STF), Pr_0.1_Ce_0.9_O_2−*δ*_ (PCO) and La_0.6_Sr_0.4_MnO_3−*δ*_ (LSM) thin films, unaltered by external degradation from contaminants, were investigated in dependence of oxygen partial pressure and temperature. This ensures that changes of the oxygen exchange kinetics can be directly related to the measurement conditions, in contrast to measurements in conventional *ex situ* setups, where the dynamics of external degradation processes also have to be considered in any mechanistic discussion. Beyond unveiling the astonishing capabilities of these materials regarding their intrinsic catalytic activity, this study emphasizes the similarities of many SOFC cathode materials with regard to the rate determining step of the OER, which seems to involve molecular oxygen and at least one charge transfer before or during the rate limiting step in incorporation direction. Moreover, the exchange rate is tightly correlated with oxygen surface vacancies. In the course of this analysis, the experimental results are discussed on the basis of an exemplary mechanism. The possibilities and limits of a reaction mechanism analysis based on electrochemical measurements are explored.

## 2. Experimental

### 2.1. Preparation of half-cell substrates

Prior to MIEC electrode deposition, platinum grids (5 μm grid width, 10 × 10 μm^2^ free squares, 100 nm thickness) were prepared by lift-off photolithography and metal sputtering (BalTec MED 020, Leica Microsystems GmbH, Germany) on both sides of (100) oriented yttria stabilized zirconia (YSZ, 5 × 5 × 0.5 mm^3^, 9.5 mol% Y_2_O_3_, Crystec GmbH, Germany) single crystalline substrates. This buried metallic grid acts as a current collector ensuring homogenous polarization of the MIEC thin film.^39^ For the substrate used during the LSM deposition, the thickness of the grid was increased to 300 nm to ensure that only the electrode area within the grid free regions was electrochemically active. The exact size of the grid-free area was measured with an optical microscope.

On the back side of the single crystals, a 200 nm thick nano-porous LSC counter electrode was applied *via* PLD (9000 pulses, 0.4 mbar *p*(O_2_), 450 °C, 1.1 J cm^−2^, 5 Hz, 5 cm target–substrate distance). This leads to electrodes with an exceptionally low polarization resistance and thus to suitable counter electrodes for the highly active thin films grown during i-PLD.^40,41^ All PLD depositions were performed with a KrF (*λ* = 248 nm) excimer laser (Lambda Physics, COMPex Pro 201). After the deposition of the counter electrode, the sample was cooled to room temperature and the edges of the samples were ground to avoid short circuits due to thin film remnants of Pt or LSC. These samples served as the basis for the subsequent i-PLD studies, using different dense oxides as model thin films for reaction mechanism analysis.

### 2.2. *In situ* impedance spectroscopy during pulsed laser deposition

The basics of this technique and the setup are described in detail in earlier studies.^36–38,42,43^ In this study a customized heating stage (Huber Scientific, Austria) for the PLD chamber was employed.^42^ The sample is placed directly on an alumina heater which is brushed with platinum paste. The counter electrode is contacted directly *via* this platinum paste. Before MIEC thin film deposition, the Pt grid on top was contacted with a Pt/Ir needle to ensure that the whole sample surface was equally polarized. The targets used during the deposition were carefully ground and preablated for 1 min with 5 Hz and 1 min with 2 Hz. The chamber was pre-evacuated below 10^−4^ mbar and the desired oxygen partial pressure during deposition (0.04 mbar) was adjusted afterwards. The temperature was controlled by measuring the (ohmic) high frequency offset resistance of the impedance curve, which is a convolution of the temperature dependent ionic resistance of YSZ, the resistance of the Pt grid and the wiring resistance of the setup. The setup resistance was measured to be 1.5 Ω for the temperatures used in this study and the Pt grid resistance was estimated based on the grid geometry.^37^ In combination with the known temperature dependence of the ionic conductivity of YSZ,^44^ this method facilitates a very precise adjustment of the substrate temperature and fluctuations can be observed directly in the impedance curves.

At the beginning of every experiment, a 40 nm thick LSC layer was deposited onto the substrate (mixed ionic/electronic conductor with >1000 S cm^−1^ electronic conductivity^45^) to ensure sufficient in-plane conductivity for the subsequent layer. The stoichiometry of all thin films was previously checked with inductively coupled plasma-mass spectroscopy and did not deviate significantly from the intended stoichiometry. All working electrode depositions in this study were performed at a substrate temperature of 600 °C and an oxygen partial pressure of 0.04 mbar, a laser frequency of 2 Hz and a fluence of 1.1 J cm^−2^. After LSC deposition, the material of interest was deposited on top and impedance spectra were recorded every 100 pulses which corresponds to a material dependent layer thickness of 1.6 to 5 nm (all growth rates were determined with reference depositions, which were examined by a profilometer (DekTakXT, Bruker, USA)). Impedance measurements were performed with an Alpha-A High Performance Frequency Analyzer and Electrochemical Test Station POT/GAL 30 V/2 A setup by Novocontrol Technologies in the frequency regime from 10^6^ to 10^−1^ Hz, a resolution of 5 points per decade and an AC RMS voltage of 10 mV. All depositions were performed at the same conditions and the deposition was stopped when changes in the polarization resistance of the electrode after additional 100 pulses became negligible (see Table 1, equilibrium thickness). The electrode capacitance increases linearly with growing film thickness, indicating its nature as volume dependent chemical capacitance. In contrast to earlier studies,^37,38^ the thickness dependence of the electrochemical properties was not of interest here. Rather, the *p*(O_2_) and *T* dependence of the polarization resistance of the surface-limited oxygen exchange reaction for equilibrium thickness was investigated in detail.

**Table 1:** Growth rates and equilibrium thickness values for the deposited materials

Material	Growth rate	Equilibrium thickness
La_0.6_Sr_0.4_CoO_3−*δ*_	33 pulses per nm	40 nm
La_0.6_Sr_0.4_FeO_3−*δ*_	71 pulses per nm	20 nm
(La_0.6_Sr_0.4_)_0.98_Pt_0.02_FeO_3−*δ*_	77 pulses per nm	25 nm
SrTi_0.3_Fe_0.7_O_3−*δ*_	60 pulses per nm	16 nm
Pr_0.1_Ce_0.9_O_2−*δ*_	18 pulses per nm	80 nm
La_0.8_Sr_0.2_MnO_3−*δ*_	40 pulses per nm	38 nm

## 3. Results

### 3.1. Growth of different SOFC cathode materials

**Fig. 1:**
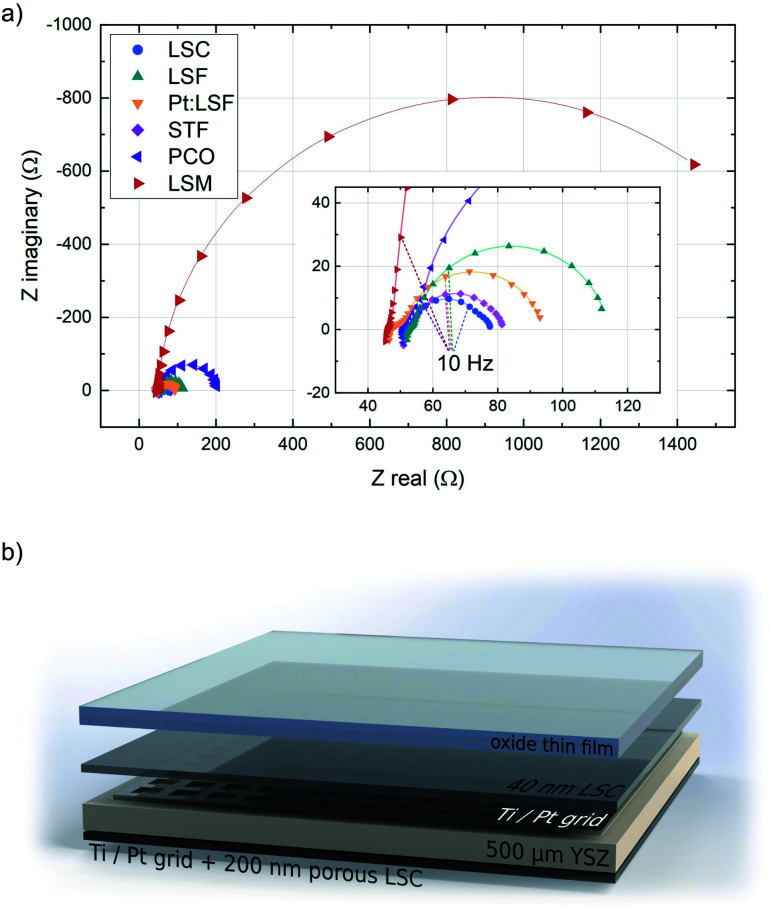
(a) Impedance curves of LSC, LSF, Pt:LSF, STF and LSM after reaching the equilibrium thickness. Spectra were recorded at 600 °C and 0.04 mbar *p*(O_2_). (b) Structure of a typical sample used during i-PLD measurements.

The initially deposited LSC layer always grew in a columnar way on the YSZ substrate. This morphology was transferred to the actual electrodes, whose surface structure was checked with AFM. The structure of the polycrystalline thin films was checked with XRD, however no clear preferential orientation across all thin films was observed. The surface exchange resistance of the initially deposited LSC layer was always between 1.2 and 1.9 Ω cm^−2^, a value in good agreement with previous *in situ* studies on LSC.^37,38^ In the impedance spectra recorded during LSC deposition, the semicircle assigned to the counter electrode is initially well separated, which allows the determination of resistance and capacitance of the porous LSC counter electrode. Those values were then fixed during the analysis of all other experiments. All materials exhibit a different equilibrium thickness, after which the surface exchange resistance remains constant during further deposition steps (see Table 1). Fig. 1 shows the impedance spectra of the different materials upon reaching this equilibrium resistance.

All impedance spectra of samples with equilibrium film thickness consist of the same basic impedance features. The ohmic offset corresponds to the ionic conductivity of the YSZ single crystal, the resistance of the Pt grid and setup impedances. At very high frequencies (>10 kHz), an inductive contribution from the wiring occurs. Also at high frequencies (10 kHz > *f* > 100 Hz), a small feature appears in most experiments (<0.1 Ω cm^−2^), which is assigned to an interfacial resistance between LSC and YSZ and a corresponding double layer capacitance.^46^ The feature of interest, which corresponds to the oxygen exchange resistance, is observed at frequencies below ∼100 Hz and is coupled with the sum of the chemical capacitance of 40 nm LSC and the top material (see ESI S.I.1†). In the case of LSC and STF (having the lowest resistance), another feature can be detected at the low frequency end of the impedance curve, which corresponds to the oxygen exchange resistance and the chemical capacitance of the porous LSC counter electrode. For all other materials, the surface kinetics of the working electrode is slower and therefore this feature is not resolvable.

During the deposition of LSM, the surface exchange resistance seems to keep increasing linearly after reaching a critical thickness. However, this corresponds to the emerging ionic diffusion resistance across the LSM layer and can be quantified from the slope of a resistance *vs.* thickness plot and subtracted to reveal the true surface exchange resistance of LSM. From the equilibrium resistances of all other materials it is apparent that they exhibit exceptionally fast oxygen reduction kinetics in their pristine state (in some cases orders of magnitude faster than literature values^46–48^), generalizing earlier findings on LSC^36,38^ to other materials. The fastest kinetics at 600 °C and 0.04 mbar *p*(O_2_) was observed on LSC (1.73 Ω cm^−2^) and on STF (1.90 Ω cm^−2^). The reason for the superior activity of pristine electrode materials has been investigated previously, indicating that the oxygen exchange kinetics experience a severe reduction upon re-heating in any *ex situ* setup due to minimal traces of sulphur in the atmosphere.^38,43^ This effect not only impairs the activity of electrode materials but also obscures mechanistic analyses of oxygen exchange on oxide surfaces (see ESI S.I.2†).

### 3.2. *p*(O_2_)- and *T*-dependence of surface exchange rates

**Fig. 2:**
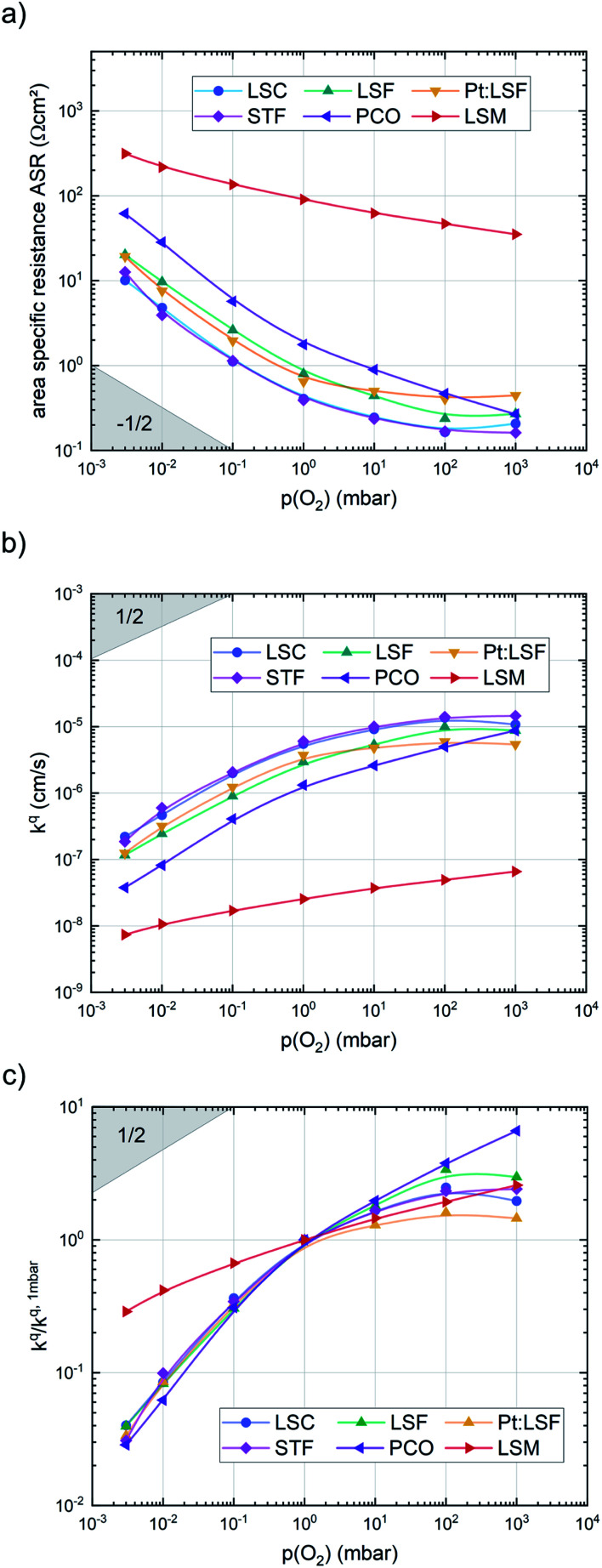
*p*(O_2_) dependence of (a) the area specific resistance, (b) the surface exchange coefficient and (c) the surface exchange coefficient, normalized to its value at 1 mbar, of pristine LSC, LSF, Pt:LSF, STF, PCO and LSM thin films at 600 °C.

After the deposition, the area specific resistance (ASR) of all materials was measured in the PLD chamber (“*in situ*”) at different oxygen partial pressures ranging from 0.003 mbar to 1000 mbar *p*(O_2_). The results are shown in Fig. 2a). The superb oxygen exchange activity measured at deposition conditions also remains at higher oxygen partial pressures, where all materials apart from LSM exhibit area specific resistance values below 1 Ω cm^−2^ at 1000 mbar and 600 °C, with LSC and STF being the fastest investigated materials with ∼0.2 Ω cm^−2^. This demonstrates the outstanding catalytic capabilities of the investigated dense layers and shows that the low resistances at deposition conditions can in principle be transferred to higher oxygen partial pressures as well.^43^

The measurements further revealed that all materials apart from LSM show a very similar correlation between ASR and *p*(O_2_). Below 1 mbar all materials exhibit a slope of 0.63 ± 0.05 and deviate from this slope at oxygen partial pressures higher than 1 mbar, where a weaker *p*(O_2_) dependence is found. The slope in this regime is hard to determine for all materials except PCO, as the surface exchange resistance slowly increases with time at high *p*(O_2_), indicating beginning surface degradation (for PCO, the slope changes from 0.68 to 0.27). This degradation is partially reversible at low oxygen partial pressure, hence backwards recorded curves contain inaccuracies at lower *p*(O_2_) (more information is presented in S.I.3†). As the atmosphere inside the PLD chamber is very clean, we assume, that a large part of these degradation phenomena at higher pressures is related to Sr migration to the surface due to intrinsic inequilibria in the crystal lattice (details of segregation mechanisms have been investigated by Niania *et al.*^30,31^), which also explains the slight differences between the materials, as different segregation dynamics in different materials can be expected.^49^Fig. 2b and c show different quantities derived from the ASR, on the one hand, the surface exchange coefficient *k*^q^ which was calculated according to eqn (2)^50^ and on the other hand, the surface exchange coefficient normalized to its value at 1 mbar, *k*^q^/*k*^q,1 mbar^, which will be used from now on to compare the different materials (*R*_surf_ denotes the polarization resistance of the oxygen exchange reaction and *c*_O_ the concentration of oxygen atoms in the crystal lattice).2
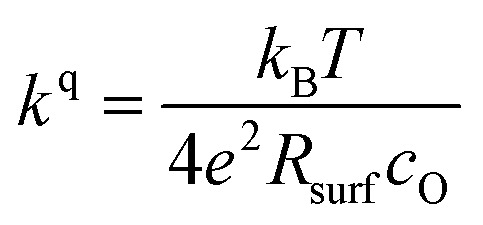


**Fig. 3:**
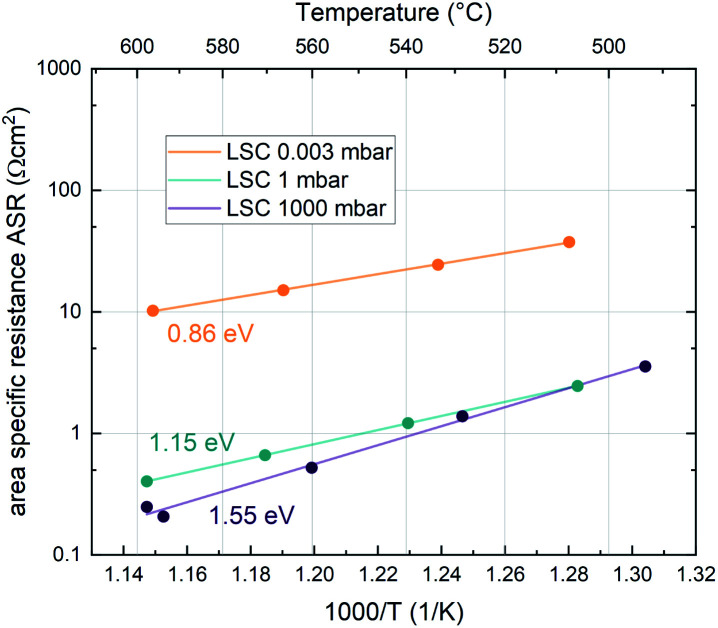
Temperature dependence of the ASR of LSC at different oxygen partial pressures. The solid lines represent Arrhenius-type fits and the corresponding activation energy is denoted next to the fit.

Additionally, the temperature dependence of the ASR was investigated at 0.003, 1 and 1000 mbar. This yields insight into the effective activation energy of the oxygen exchange reaction. An exemplary measurement on LSC is shown in Fig. 3. The effective activation energy increases continuously with rising oxygen partial pressure, from 0.86 eV at 0.003 mbar up to 1.55 eV at 1000 mbar. The measurement direction (high *T* to low *T* or *vice versa*) does not yield different results, only at high oxygen partial pressures, the effective activation energy of the oxygen exchange resistance contains some uncertainty, as degradation processes are occurring on the timescale of the measurement, slightly masking the true thermal activation (see the two data points at ∼600 °C and 1000 mbar in Fig. 3). However, it is still clear that the effective activation energy increases considerably between 1 mbar and 1000 mbar.

**Fig. 4:**
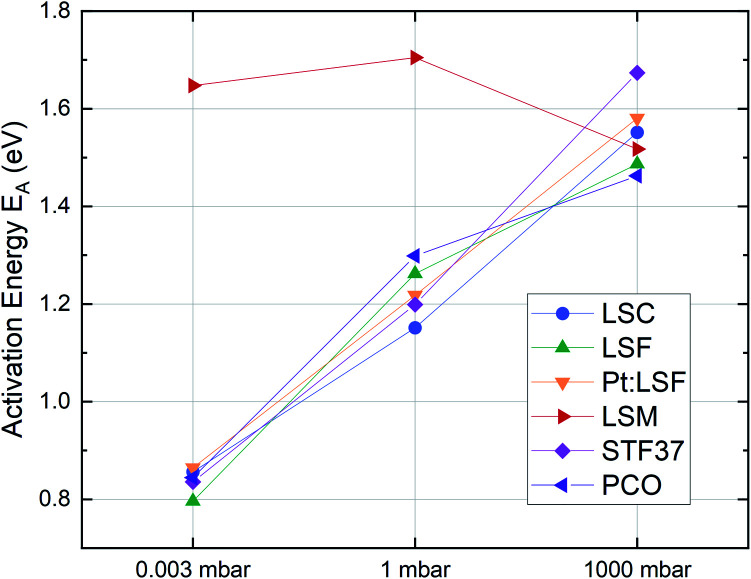
Activation energies of pristine LSC, LSF, Pt:LSF, STF, PCO and LSM thin films at different oxygen partial pressures.

The effective activation energies of all materials are plotted in Fig. 4. This figure again shows surprising similarities between all materials apart from LSM, whose activation energy shows a rather weak *p*(O_2_) dependence. For all other materials the effective activation energy increases from 0.84 ± 0.03 eV at 0.003 mbar to 1.55 ± 0.08 eV at 1000 mbar.

### 3.3. Current density – overpotential curves

To extend the investigation to non-equilibrium kinetics, bias voltage was first applied at 0.1 mbar *p*(O_2_) on pristine, uncontaminated LSF thin films inside the PLD chamber using the i-PLD setup. Whereas in the previous measurements the forward reaction rate equals the backward reaction rate, the current flows predominantly in one direction when applying overpotential (cathodic ↔ oxygen incorporation & anodic ↔ oxygen evolution). Due to the complexity of the experimental setup, it was not possible to perform 3-electrode measurements. However, the counter electrode exhibits a much higher chemical capacitance than the working electrode and at low oxygen partial pressures (0.1 mbar), the two features are well separated in an impedance measurement with anodic bias voltage (see S.I.4 in the ESI†). As the resistance measured in an impedance spectroscopic measurement represents the differential slope of a current density–overpotential curve, the overpotential dropping at the working electrode, *η*_WE_ can be calculated by integrating the measured working electrode AC resistance *R*_WE_ over the DC current measured in the experiment:3
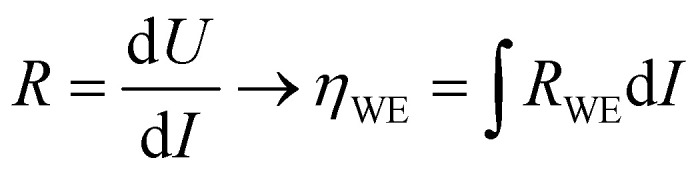


To check the validity of this method, the voltage dropping at the working electrode was calculated directly and indirectly *via* subtracting the voltage drop in the YSZ electrolyte and in the counter electrode from the total applied voltage. Both methods differ by less than 5%, thereby confirming the consistency of this calculation.

**Fig. 5:**
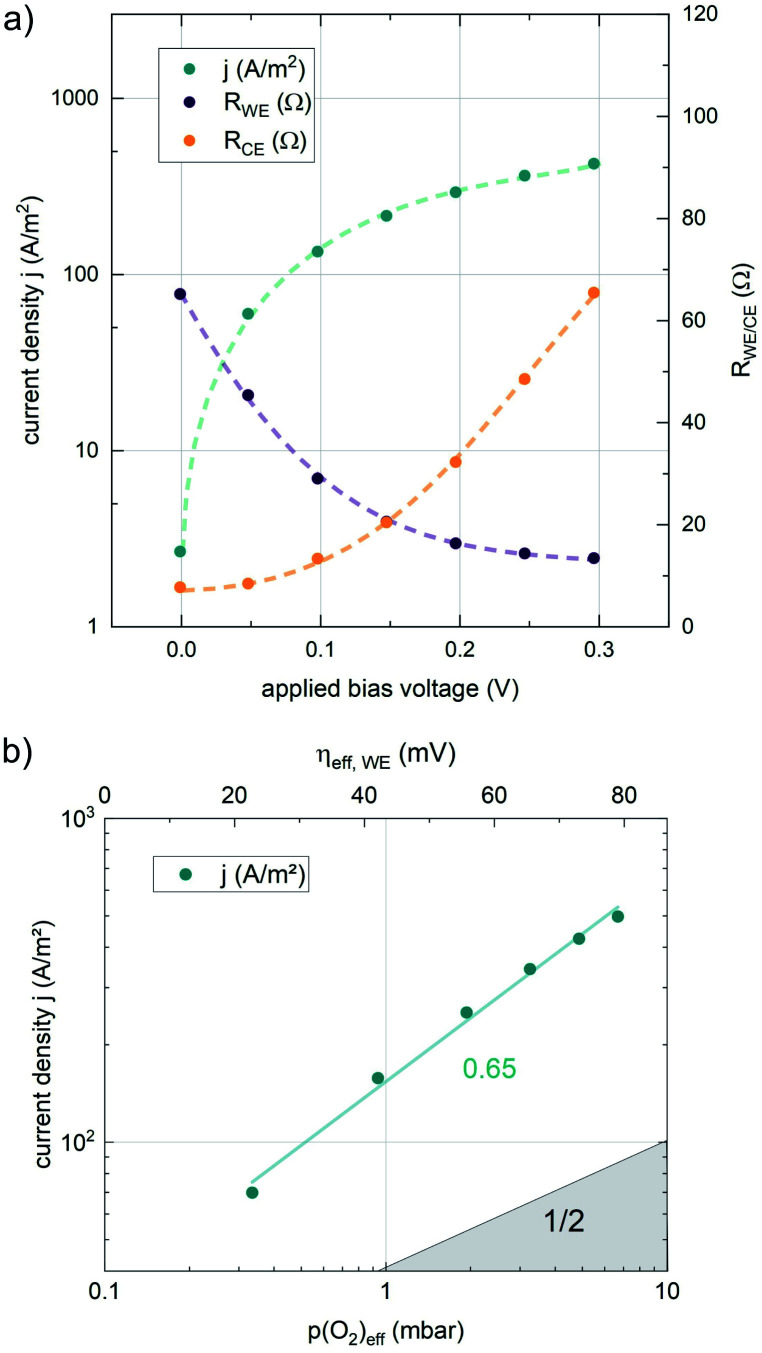
(a) Current density and resistance values of LSF working and porous LSC counter electrode under applied bias voltage at 0.1 mbar *p*(O_2_) and 600 °C. (b) Current density at the working electrode *vs.* the effective *p*(O_2_) generated by the bias voltage drop inside of the working electrode, calculated *via* the Nernst equation for a true (gas) partial pressure of 0.1 mbar *p*(O_2_).

The results of anodic polarization measurements are shown in Fig. 5a). While the AC resistance of the LSF working electrode, *R*_WE_, decreases, the resistance of the counter electrode *R*_CE_ increases continuously, possibly indicating the onset of a transport limitation. Nevertheless, the two well separated features allow the investigation of the correlation between the resistance of the working electrode and the overpotential at the working electrode. This overpotential can be translated into an oxygen chemical potential in the electrode and, *via* Nernst's equation, into an effective oxygen partial pressure or a specific defect chemical situation in the working electrode material. In a similar partial pressure range where *k*^q^ shows a real oxygen partial pressure dependence of ∼0.63, also the current density of the oxygen evolution reaction plotted *vs.* the effective *p*(O_2_) within the MIEC electrode in a log–log diagram exhibits a slope of 0.65 (see Fig. 5), indicating consistency of our analysis (assuming only minor contributions from adsorbates, as these could affect effective and real *p*(O_2_) dependencies differently).

**Fig. 6:**
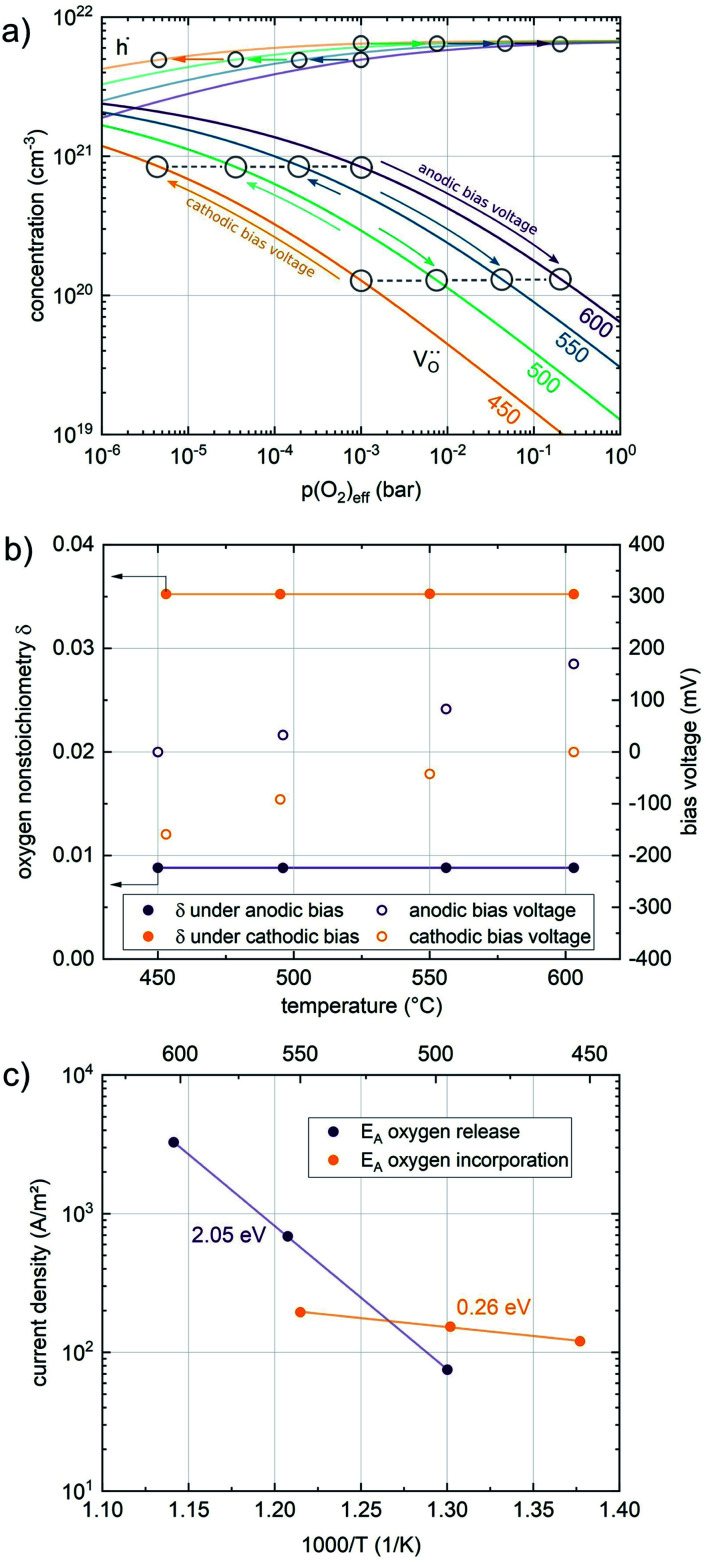
(a) Calculated concentrations of oxygen vacancies and electron holes in LSF between 450 and 600 °C. Circles indicate oxygen partial pressures where the oxygen vacancy concentration is the same at different temperatures, arrows represent the bias voltage required to achieve this effective oxygen partial pressure for a measurement atmosphere of 1 mbar *p*(O_2_). (b) Actual oxygen nonstoichiometry measured at different temperatures and the bias voltage required to obtain these values. (c) Resulting concentration amended activation energies for the oxygen evolution and the oxygen incorporation reaction.

Nonequilibrium measurements can also be used to elucidate hidden aspects of the activation energy of the oxygen exchange reaction. Since the defect chemistry of the investigated materials is strongly temperature dependent (more details in the discussion), the effective activation energy observed in equilibrium measurements is actually a convolution of the “true” reaction barrier and other contributions, especially from temperature dependent defect concentrations. Therefore, a novel measurement technique is introduced here to unravel this convolution. Measurements under anodic as well as cathodic bias voltages on LSF thin films were performed at 1 mbar *p*(O_2_) and different temperatures and the bias voltage was used to control the defect concentrations in the material and to fix them to a certain level for different temperatures, to access concentration amended activation energies (see Fig. 6a).

For measurements under anodic bias, the chemical capacitance *C*_chem_ of an LSF thin film was first measured at 450 °C and the oxygen nonstoichiometry corresponding to this capacitance was calculated assuming a dilute defect model according to the method described in ref. 38. In the following, anodic bias voltage was applied to the LSF thin film, whereby the effective oxygen partial pressure in the material is increased and the oxygen vacancy concentration is reduced. For 500, 550 and 600 °C, the exact bias voltage was determined, where the oxygen vacancy concentration, again calculated from *C*_chem_, equalled the initially measured value at 450 °C. The same procedure was performed for cathodic bias voltages, starting in equilibrium at 600 °C. Here, increasing the applied cathodic bias voltage for lower temperatures leads to lower effective oxygen partial pressures, more oxygen vacancies and thus a higher chemical capacitance. The applied voltage was again increased until the desired oxygen vacancy concentration was reached. With this method, the concentration dependent term of the effective activation energies of the oxygen incorporation and evolution reactions could be eliminated (the oxygen vacancy concentration is actively kept constant, differences in the electron hole concentration are negligible). The calculated oxygen nonstoichiometry is shown in Fig. 6b. This yields concentration amended activation energies of 0.26 eV for the oxygen incorporation reaction and 2.05 eV for the oxygen evolution reaction (see Fig. 6c), which are both drastically different from 1.25 eV, the effective activation energy of LSF during equilibrium measurements at 1 mbar *p*(O_2_). To the best of our knowledge, such concentration amended activation energies have not been deduced so far for the oxygen exchange reaction on mixed conducting oxides.

## 4. Discussion

### 4.1. General remarks

The similarities of LSC, LSF, Pt:LSF, STF and PCO strongly suggest, that all these materials share a similar reaction mechanism in their pristine state. LSM is known to exchange oxygen primarily either at the triple phase boundary or *via* grain boundaries and is expected to behave different to the other materials.^51–53^ This can also be observed during i-PLD. For this reason, LSM will be omitted from the further discussion. In the following, the key experimental observations and their implications for the OER mechanism of the investigated materials will be discussed on the basis of an exemplary mechanism which satisfies all constraints given by the measurement results:

(a) The oxygen exchange kinetics of pristine LSC, LSF, Pt:LSF, STF and PCO exhibit a very similar qualitative behaviour upon *p*(O_2_) and *T* variations.

(b) All materials share a slope of ∼0.63 ± 0.05 of the surface exchange coefficient *k*^q^*vs. p*(O_2_) below 1 mbar.

(c) The slope of the *p*(O_2_) dependence of *k*^q^ decreases for all investigated materials above 1 mbar, although to a different extent for different materials.

(d) The effective activation energy of oxygen exchange increases significantly with *p*(O_2_) for all materials and its absolute value is similar for all materials (from 0.83 ± 0.03 eV at 0.003 mbar to 1.56 ± 0.10 eV at 1000 mbar).

(e) The concentration amended activation energies for oxygen incorporation and evolution are very different (0.26 *vs.* 2.05 eV for LSF at 1 mbar) while the corresponding effective activation energy amounts to ∼1.25 eV (from equilibrium measurements).

**Fig. 7:**
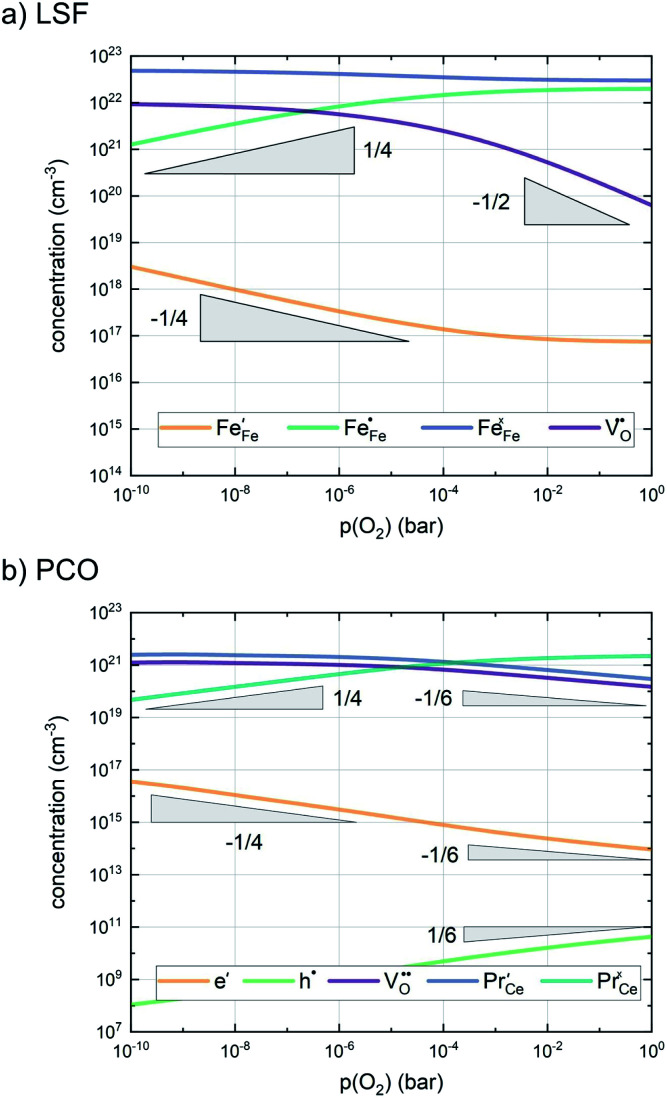
Calculated defect concentrations of LSF and PCO at 600 °C between 10^−10^ and 1 bar *p*(O_2_), calculated from literature data.^62,64,65^

In any mechanistic discussion, it is essential that all arguments are valid for the complete reaction mechanisms, *i.e.* forward and backward directions must be considered in all conclusions drawn from the measurement results. Especially with regard to the temperature dependence of the reaction rates, the two directions must be considered separately, as they may have significantly different activation barriers. Moreover, the defect chemistry of the investigated materials is a major factor in mechanistic discussions as it is directly related to temperature and partial pressure dependences of the oxygen exchange reaction rate. Among the materials investigated in this study, we assume that LSF, Pt:LSF, LSC and STF exhibit many defect chemical similarities, while PCO is governed by a fundamentally different defect chemistry. However, even this assumption includes major simplifications, as LSC is often associated with delocalized electron hole conductivity, while the defect chemistry of LSF includes electron holes localized on Fe atoms.^11,54,55^ In the absence of an appropriate analytical defect model for LSC, it is treated similar to LSF in this study. Furthermore, STF is generally viewed as an ideal solid solution of SrTiO_3_ and SrFeO_3_ and modelled with a certain amount of so-called structural vacancies, *i.e.* a defect model based on thermogravimetric measurements and with only limited suitability for the discussion of oxygen exchange kinetics.^56–59^ The defect concentrations of LSF and PCO calculated from the respective defect chemical models with mass action constants from literature are shown in Fig. 7.^60–65^ For further details on the calculation and the used mass action constants, please refer to S.I.5 in the ESI.†

To further complicate the matter, as the oxygen exchange reaction occurs at the materials surface, the concentrations of the charge carriers partaking in the reaction can differ considerably from bulk concentrations, especially with regard to oxygen vacancies.^66–69^ A simplified approach employed due to the lack of quantitative data on the surface chemistry of perovskites treats the defect chemistry of the surface as reduced or oxidized with respect to the bulk material, but maintaining electroneutrality, *i.e.* a *p*(O_2_)-displacement in the Brouwer diagram. With regard to the quantitative difference between surface and bulk defect chemistry, literature research indicates that the surface of perovskites is predominantly reduced compared to the bulk material,^67,69^ however, a quantification of this difference does not yet exist, a rough estimate is attempted later on.

### 4.2. Rate equation and suggested mechanism

To compare the experimental results with predictions from defect chemical considerations, reaction rates must be correlated with defect concentrations. According to Schmid *et al.*,^70^ the reaction rates of oxygen incorporation or oxygen evolution can often be expressed as4
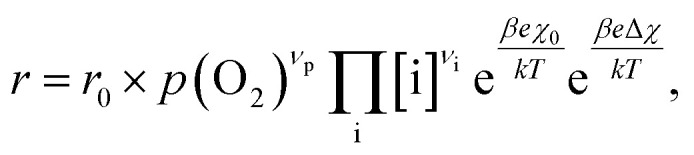
where *r* stands for the overall reaction rate, *r*_0_ is a prefactor including the reaction barrier as well as mass action constants from other reaction steps, *p*(O_2_) is the oxygen partial pressure and [i] is the concentration of defect i. *ν*_p_ and *ν*_i_ describe the respective reaction orders of *p*(O_2_) and defect i which depend on the reaction mechanism and the considered reaction direction. Please note that the *p*(O_2_) term represents the partaking oxygen species still in equilibrium with the gas phase, *i.e.* either adsorbates or even gas molecules. The corresponding exponent *ν*_p_ is thus generally 0 for the oxygen evolution reaction and 1 or 1/2 for molecular or atomic adsorbates for oxygen reduction, respectively (in dilute cases). The exponential terms describe the effect of the equilibrium surface potential *χ*_0_ and its variation under electric current, Δ*χ*, where *β* is a factor depending on the reaction mechanism and *e*, *k* and *T* represent the elemental charge, the Boltzmann constant and the temperature, respectively. Depending on the mechanism, also more complicated concentration dependences are possible, which cannot be expressed in terms of simple power laws, particularly for non-dilute situations.

When studying eqn (4) in more detail, it becomes apparent that temperature and oxygen partial pressure can affect the reaction rate in various ways. The oxygen partial pressure on the one hand directly affects the adsorbate concentration on the surface (*p*(O_2_)^*v*_p_^) and on the other hand, defect concentrations themselves are strongly *p*(O_2_) dependent, as has already been shown above. Any *p*(O_2_) dependence of the surface potential and its variation under current is neglected in this discussion, as well as any dependence on overpotential (Δ*χ* = 0). The temperature dependence of the reaction rate is tied to the exponential terms correlated to the surface potential, to the activation barrier of the reaction, to mass action equilibria and to the defect concentrations themselves, which also change with temperature according to the materials defect chemistry. The convolute of all these contributions is observed when an effective activation energy is measured in equilibrium conditions.

A first general conclusion concerning the mechanism can already be drawn from the experimentally observed slope of the reaction rate in the oxygen partial pressure regime below 1 mbar *p*(O_2_) (∼0.63). For atomic adsorbates (without site restriction), the slope of the reaction rate cannot exceed 0.5 for any reasonable mechanism (see S.I.6†), as the concentrations of all potentially included defects are either constant or lower the slope (*e.g.* for the 1/4 regime of electron holes – Fig. 7). We take this as a strong indication that molecular oxygen is involved in the reaction, *i.e. ν*_p_ = 1. However, the measured slope is also significantly less than 1. This requires some defect species to take part in the reaction, with their concentration reducing the slope of the reaction rate with regard to oxygen partial pressure. At lower oxygen partial pressures this can be caused by a contributions from electronic defects and from oxygen vacancies, whose concentration begins to decrease. At higher oxygen partial pressures, on the other hand, the experimentally observed reaction rate exhibits a strongly suppressed *p*(O_2_) dependence. Considering *e.g.* the Brouwer diagram of LSF (Fig. 7), one possible explanation for this behaviour is the partaking of oxygen vacancies in the reaction, as their concentration drops sharply with a slope of up to 0.5 at higher oxygen partial pressures. Also, an adsorbate coverage deviation from Langmuir behaviour could contribute to a decrease of the *p*(O_2_) dependence.

In the following, we first suggest a mechanism and then discuss how it explains the main features of our measurements. Naturally, this does not ultimately prove its validity, since also alternative mechanisms may explain the results. However, we believe that several key features of this mechanism are relevant for our materials.

**Fig. 8:**
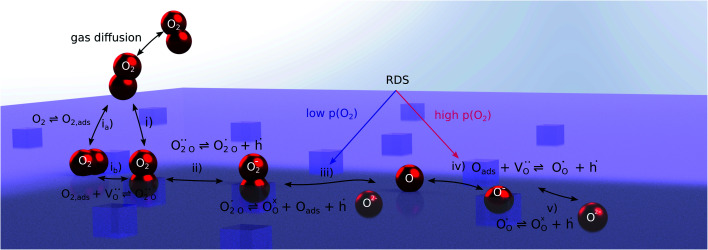
Visualization of an exemplary oxygen exchange mechanism with electron holes partaking in the charge transfer reactions. The rate limiting steps at low and high oxygen partial pressures are indicated accordingly.

The exemplary mechanism (see Fig. 8) to evaluate the i-PLD results in consideration of the previously discussed model consists of the following steps for the incorporation direction: (i) adsorption of molecular oxygen in a surface vacancy; (ii) first electron transfer to the adsorbed oxygen molecule; (iii) second electron transfer and dissociation of the adsorbed molecule; (iv) transfer of the remaining adatom into a second surface vacancy with concomitant ionization; (v) final electron transfer and incorporation into the host lattice. The electron transfer processes in this mechanism do not mean that the charge is transferred across the interface (= surface); rather it is transferred within the surface layer. Then, the *β* factor in eqn (4) becomes zero. The adsorption step could potentially also be split into a surface adsorption and subsequent transfer into a surface vacancy, i_a_) + i_b_). This step would also include surface diffusion and vacancy migration.

As long as plenty of oxygen vacancies are available (low *p*(O_2_)), assuming dissociation as rate determining step seems reasonable (step iii). As with increasing *p*(O_2_) the oxygen vacancy concentration drops considerably, we suspect that at higher oxygen partial pressures the rate limitation is correlated with the reduced availability of surface vacancies. Thus, the rate limiting steps of the reaction are step (iii) at low *p*(O_2_) (<1 mbar) and step (iv) at higher *p*(O_2_). Depending on the defect chemistry of the material, charge transfer steps can include electrons or electron holes as essential species. For LSC, LSF, Pt:LSF and STF, electron holes are assumed to partake in charge transfer steps. In LSF, for example, an electron transfer to oxygen would turn an Fe^3+^ into an Fe^4+^ ion, thereby creating a polaronic hole. For PCO, electrons (Pr^′^_Ce_) are supposed to be the relevant charge carriers.

Written as a series of chemical reactions with the according mass action laws, this mechanism reads for holes being involved:
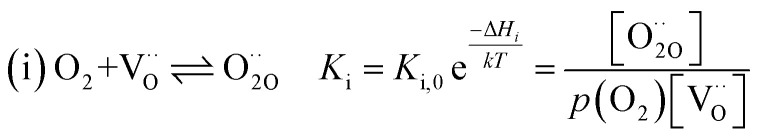

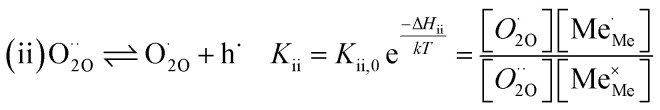

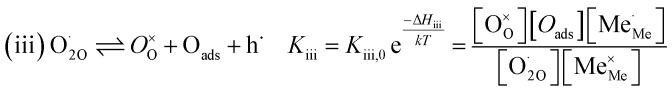

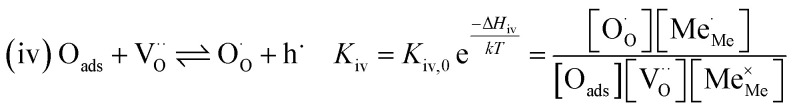

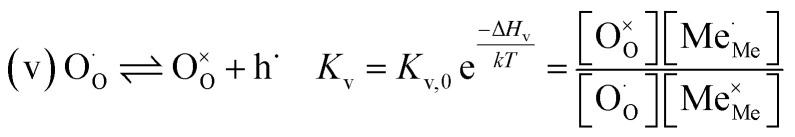
*K* and Δ*H* denote the corresponding mass action constant and reaction enthalpy. To predict the according reaction rates from defect chemical calculations, it is necessary to correlate the reaction rate to defect concentrations, as discussed above. This leads to the following rate equations and concentration amended activation energies for the two rate limiting steps, which are used to predict the reaction rate from defect concentrations:

RDS at low *p*(O_2_) − step (iii):5
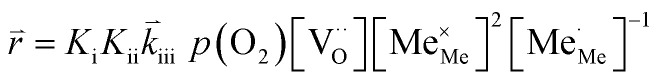
6
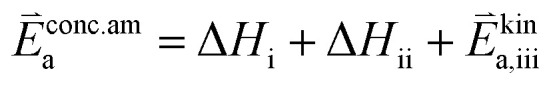
7
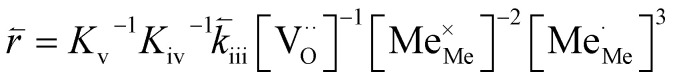
8
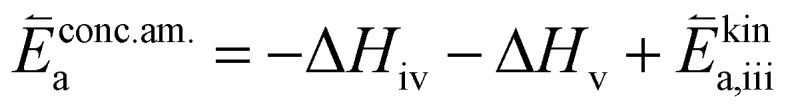


RDS at high *p*(O_2_) – step (iv):9
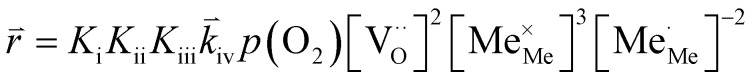
10
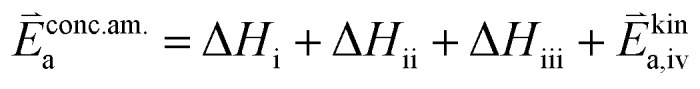
11
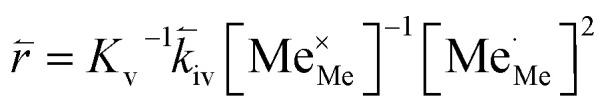
12
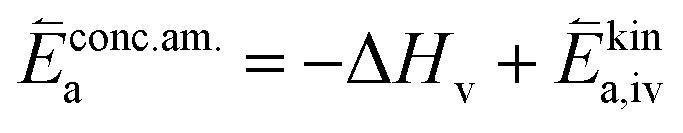


In these rate equations all relevant mass action constants with their respective temperature dependences are included, as well as a rate constant *k* including the chemical activation barrier of the rate determining step *E*^kin^_a_. Moreover the rate equations consider the oxygen partial pressure and the fundamental defect concentrations. It also further clarifies the difference between the chemical activation barrier *E*^kin^_a_, the concentration amended activation energy 
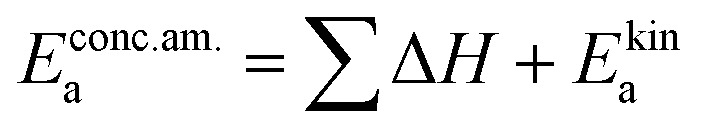
 and the effective activation energy which also includes all defect concentration related temperature dependences. Terms related to the surface potential are omitted, as *β* amounts to 0 as discussed above.

### 4.3. *p*(O_2_)-dependent reaction rates

**Fig. 9:**
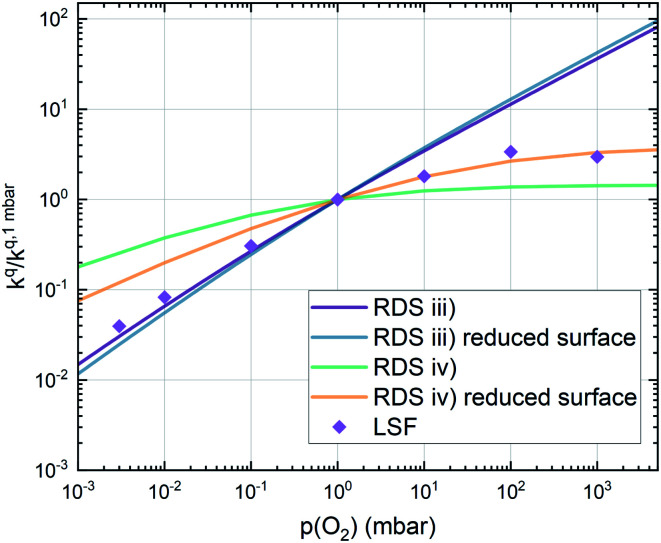
Predicted oxygen surface exchange coefficients of LSF for both rate limiting steps (iii) and (iv) as well as for each rate limiting step with a reduced surface, achieved by a shift in the Brouwer diagram of 175 meV. Symbols represent experimental values for an LSF thin film.

Combining the calculated defect concentrations of the respective materials defect model with the rate model described above, it is possible to predict the *p*(O_2_) dependence of the measured reaction rates. Fig. 9 shows the calculated reaction rates of LSF for both of the aforementioned rate determining steps. The reaction rate at low oxygen partial pressures is well estimated with RDS (iii) while at high *p*(O_2_) this rate determining step severely overestimates the reaction rate. Conversely, RDS (iv) achieves a better fit at higher oxygen partial pressures, slightly underestimating the experimental values, while it yields a poor representation at lower *p*(O_2_). The possibility of a reduced surface, as discussed above, can be included in the calculations by applying a shift in the Brouwer diagram to lower oxygen partial pressures. This has a minor effect on the reaction rate of RDS (iii) and a significant impact on RDS (iv). The best fit of the experimental reaction rates was achieved with a chemical potential shift of 175 meV for e^−^ and h˙, corresponding to a partial pressure shift of the surface defect chemistry by two orders of magnitude. The specific slope of ∼0.63 at low *p*(O_2_) is thus essentially the consequence of *ν*_p_ = 1 in eqn (5), the decreasing electron hole concentration with increasing *p*(O_2_) (*ν*_h_ at most −0.25) and the onset of the decreasing oxygen vacancy concentration. The smaller *p*(O_2_) dependence at higher *p*(O_2_) is a counterbalancing of *ν*_p_ = 1 for gas molecules or O_2_ adsorbates and *ν*_V_ = 2 with 
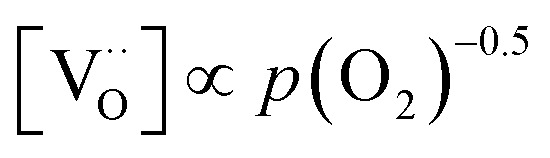
 at most.

**Fig. 10:**
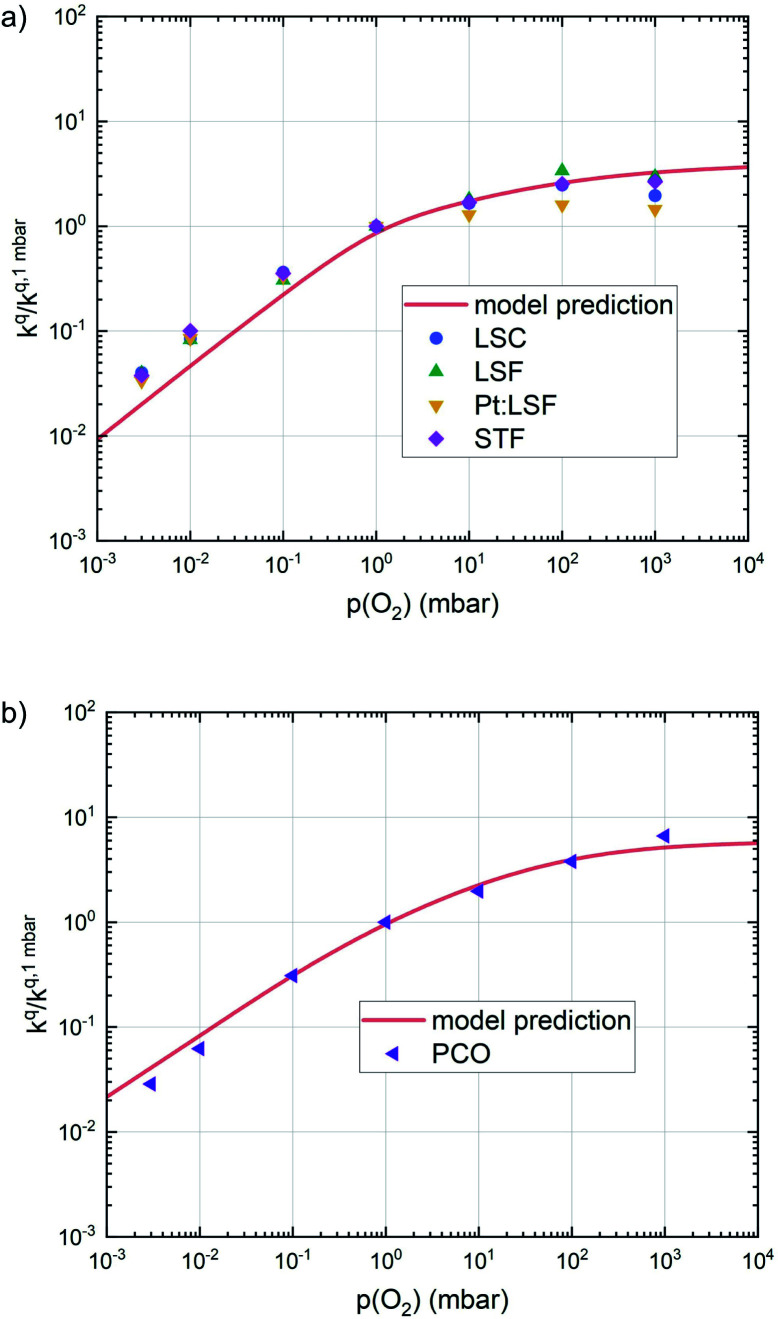
Oxygen surface exchange coefficients on perovskite materials (a) and on PCO (b) from equilibrium measurements plotted together with the prediction from defect chemical calculations.

This analysis indicates, that a mechanism with only one unique rate determining step cannot explain the experimentally observed reaction rates and that the defect chemical predictions of the chosen mechanism with the transition between the two rate determining steps and a slightly reduced surface achieve a good agreement with the experimental data. Extending this analysis to PCO, where a different defect model needs to be considered, shows that also here this interpretation of the experiments agrees very well with the experimental results. Interestingly, also the observed reaction rates of the other perovskite materials fit very well to the calculated reaction rate of LSF (see Fig. 10). We take this as a strong indication that all materials indeed share a common reaction mechanism and that the *p*(O_2_) dependences of defect concentrations share surprising similarities (especially with regard to the position of the transition towards lower oxygen vacancy concentrations in the Brouwer diagram). In conclusion, we believe that the proposed mechanism is well suited to reflect the key aspects of the oxygen surface exchange on these materials.

The discussion so far is further reinforced by the performed polarization experiments. During measurements far from equilibrium, only one reaction direction governs the reaction. In the case of measurements under cathodic polarization, this is the oxygen incorporation, for anodic polarization, it is the oxygen evolution reaction. In our model, the reaction rate is only affected by bias induced changes of the defect concentrations of the material.^71^ The good agreement between the slopes measured during equilibrium experiments and anodic polarization experiments (see Fig. 5) further confirms the results, as for the oxygen evolution reaction, the slopes of experiments with real *p*(O_2_) changes and of experiments with effective *p*(O_2_) variation induced by bias voltage should coincide.

### 4.4. Activation energies

The activation energy of the surface exchange reaction requires a more sensible treatment and is a widely discussed issue in literature concerned with oxygen exchange mechanisms. Referring to the discussion above, a key aspect has already been pointed out, namely that multiple factors affect the temperature dependence of the reaction rate, *e.g.* mass action equilibria, the reaction barrier *E*^kin^_a_, the surface potential (if *β* ≠ 0) and defect concentrations (*cf.*eqn (5)–(12)). Solely based on equilibrium measurements of the temperature dependence of the reaction rate, where only the effective activation energy is measured, it is therefore generally not possible to isolate a true reaction barrier from other contributions to an effectively measured Arrhenius dependence. Moreover, it is very helpful to consider both reaction directions. While the effective activation energy in equilibrium conditions must be the same for both reaction directions, individual contributions to this activation energy like the reaction barrier *E*^kin^_a_ or defect chemical contributions can be (and are expected to be) drastically different for the two directions.

To give a first estimate for the impact of the defect chemical contributions, the temperature dependence of defect concentrations was calculated for LSF and a purely defect concentration related activation energy was calculated for exemplary rate determining steps (see S.I.7†). This evaluation shows that for the oxygen incorporation direction, this contribution is positive (between 0.5 and 2 eV) and increases continuously over a wide *p*(O_2_) window. For the oxygen evolution reaction, this defect concentration related activation energy also increases with *p*(O_2_), however, it is always negative (between 0 and −1.5 eV). This analysis illustrates how essential it is to consider the materials defect chemistry when discussing activation energies.

The concentration amended activation energies observed in bias experiments are isolated from defect chemical contributions and only include the actual reaction barrier of the rate determining step *E*^kin^_a_ and reaction enthalpies in the prefactor Δ*H* (see eqn (5)–(12)). While the large difference between the concentration amended activation energies of the two reaction directions (0.26 eV for oxygen incorporation and 2.05 eV for oxygen evolution) may seem surprising at first, such a difference could have already been suspected due to the vastly different defect chemical contributions of the two reaction directions.

Apart from general statements about the activation energies of the oxygen exchange reaction, a more detailed evaluation of the experimental results also yields further information in the present mechanistic discussion. Owing to experimental reasons, the determination of concentration amended activation energies is limited to a very small oxygen partial pressure range and the values determined refer to 1 mbar where we have a mixed regime of two rate limiting steps. Activation energies for both rate limiting steps of the proposed mechanism and both reaction directions at 1 mbar were calculated. The activation energies of the defect concentrations in eqn (5), (7), (9) and (11) from the defect chemical model were calculated (as shown in S.I.7†) and the arithmetic mean for the two rate determining steps was added to the concentration amended activation energies of oxygen incorporation and oxygen evolution, again assuming a reduced surface (corresponding to a chemical potential shift of 175 meV). This analysis yields an effective activation energy of 1.44 eV for the incorporation reaction at 1 mbar and 1.29 eV for the oxygen evolution reaction, both being in rather good agreement with the effective activation energy of 1.26 eV measured during equilibrium measurements, thus strongly supporting all previous conclusions and the choice of the proposed mechanism. Accordingly, the effective activation energy of the oxygen incorporation direction mainly consists of the large activation energy contribution of oxygen vacancies. Importantly, it rules out any mechanisms including no oxygen vacancies before or in the rate determining step in the incorporation direction at these oxygen partial pressures. It also becomes clear that, while this analysis brings us a significant step towards the true energetic barrier of the rate determining reaction steps, the concentration amended activation energies still contains further enthalpic contributions which cannot be isolated from *E*^kin^_a_.

To further understand the origin of the measured activation energies, it is helpful to view these results in the context of the specific reaction steps (Fig. 10). At high oxygen partial pressures, the rate limiting step is the incorporation of an oxygen adatom into an oxygen vacancy with a concomitant ionization or the release from said vacancy in the backwards reaction direction (reaction rates given by (9) and (11) respectively). The much higher concentration amended activation energy measured in the oxygen evolution direction agrees with chemical intuition, as the oxygen atom needs to break its bonds to the surrounding surface atoms and proceed to an energetically less favourable adsorbed state on the surface (−Δ*H*_iv_ and thus also 
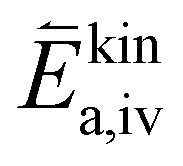
 are large). While at low oxygen partial pressures the actual kinetic reaction barrier 
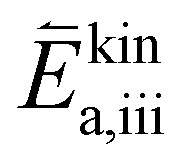
 might not be as high, one must also consider the species concentrations during the previous reaction steps. For this reason, also here, the oxygen evolution reaction will exhibit a much larger total activation energy, as the species before the rate limiting step will only be available in low concentration (Δ*H*_iv_ is still large).

### 4.5. Differences & similarities of the investigated materials

Apart from discussing mechanistic similarities of the oxygen exchange reaction on SOFC cathode surfaces, the experimental results also encourage to discuss the quantitative differences between the reaction rates of the various investigated materials. While it is not possible to unambiguously identify their origin, we will point out some possibilities which could account for the observed differences. For the rate limiting steps considered in this study, several factors affect the total rate of the oxygen exchange. A significant material dependence is expected particularly for adsorption thermodynamics (*K*_i_), the availability of oxygen surface vacancies and the ease of charge transfer to the adsorbed oxygen (*K*_ii_ and 
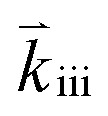
). All of these may cause differences between the materials. For PCO, a combination of different oxygen vacancy concentrations and lower electron transfer rates might contribute to the lower reaction rate. For STF, it was already mentioned above, that the defect model is based on an ideal solid solution and structural vacancies are used to model the defect chemistry. Although this complicates any predictions of reaction rates, we expect that the relevant oxygen vacancy concentration could be substantially higher than in LSF which might increase the reaction rate. For Pt:LSF, the Pt doping possibly also increases the oxygen vacancy concentration leading to increased reaction rates (Pt was determined to be present as Pt^4+^, more details about the properties of Pt:LSF are presented in a separate paper). However, it is again worth mentioning that the qualitative behaviour is astonishingly similar and as discussed before, indicates not only a similar reaction mechanism but also that the defect chemistry of all investigated materials exhibits similar changes in the observed *p*(O_2_) range. Other factors affecting the absolute reaction rate could originate from the charge transfer steps which may work differently in the specific materials (different transition metal cations or lattice oxygen may play an important role in the formation of ionized oxygen molecules on the surface^72^) and, additionally, the surface chemistry of the materials is expected to differ from bulk defect concentrations, adding another layer of complexity (also different surface reconstructions could play a role, as they may stabilize different surface chemistries).^73,74^ To further elucidate the last aspect, detailed studies of the surface chemistry of the investigated materials are required.

## 5. Conclusions

The oxygen surface exchange rate on pristine, uncontaminated, dense LSC, LSF, Pt:LSF, STF, PCO and LSM thin films was determined with *in situ* impedance spectroscopy during pulsed laser deposition (i-PLD) in a *p*(O_2_) range between 0.003 mbar and 1000 mbar and a temperature range from 500 to 600 °C. As first result, very low polarization resistances (∼0.2 Ω cm^−2^ for LSC and STF) were observed at 1000 mbar *p*(O_2_). Second, all materials except LSM show a very similar *p*(O_2_) dependence of the reaction rate, indicating a similar oxygen exchange mechanism on the different materials. All materials exhibit a similar slope of around 0.63 below 1 mbar and a weaker *p*(O_2_) dependence above 1 mbar. This flattening is a convolution of slight degradation effects and a real *p*(O_2_) dependence of the oxygen surface exchange rate. Furthermore, the activation energy of the surface exchange resistance was measured at different oxygen partial pressures, revealing a continuous increase of the activation energy with rising *p*(O_2_). To extend the investigation to non-equilibrium conditions, polarization experiments were performed on pristine LSF thin films. These measurements revealed a 0.65 slope of the anodic current density *vs.* the effective *p*(O_2_) induced by the overpotential, being in excellent agreement with equilibrium measurements. Moreover, it was possible to eliminate concentration related contributions to the effective activation energies of oxygen incorporation and evolution at 1 mbar by controlling the materials defect chemistry at different temperatures *via* the application of appropriate bias voltages. This revealed significantly different concentration amended activation energies for the oxygen incorporation and oxygen evolution reaction on LSF (0.26 *vs.* 2.05 eV). The results of these measurements pose a set of constraints on the mechanism of the oxygen exchange reaction and a mechanism is proposed which satisfies these constraints and, in combination with defect chemical predictions, is in excellent agreement with all experimental results. The rate limiting steps of this mechanism are the dissociation of an oxygen molecule adsorbed in an oxygen vacancy at low *p*(O_2_) and the incorporation of the resulting oxygen adatom in a second oxygen vacancy at higher *p*(O_2_). It became clear that the *p*(O_2_) dependence of the reaction rate (*e.g.* 0.63 slope at low *p*(O_2_)) is caused by a combination of molecular adsorbates in a vacancy and *p*(O_2_) dependent defect concentrations. Also, activation energies are shown to be a convolute of numerous thermodynamic and kinetic contributions and are strongly affected by the temperature dependence of defect concentrations. Even concentration amended activation energies are not straight-forward to explain, although the very high value for the oxygen evolution reaction (2.05 eV) may particularly arise from the barrier for oxygen atoms leaving the lattice. The results of this study strongly emphasize that any discussion of reaction mechanisms of the oxygen surface exchange reaction requires detailed information on the defect chemistry of the investigated materials, especially with regard to their surfaces.

## Conflicts of interest

There are no conflicts of interest to declare.

## Supplementary Material

TA-010-D1TA07128A-s001
