# Supplementary material for: Investigating oxygen reduction pathways on pristine SOFC cathode surfaces by in situ PLD impedance spectroscopy
Source: J Mater Chem A Mater. 2021 Nov 5;10(5):2305–19. doi: 10.1039/d1ta07128a (PMC8805794; doi:10.1039/d1ta07128a)
Supplement: TA-010-D1TA07128A-s001 [file TA-010-D1TA07128A-s001.pdf]

Electronic Supplementary Material (ESI) for Journal of Materials Chemistry A.
This journal is © The Royal Society of Chemistry 2021

Supporting Information

S.I.1 Chemical Capacitances during i-PLD measurements

The chemical capacitances of the deposited thin films have been estimated from impedance spectra. The capacitance of the LSC sublayer has been subtracted from the total capacitance of the thin film. However, we suspect that during the deposition of multilayer structures, sublayers can be subject to substantial strain from a deposited top layer. Therefore, chemical capacitances may be afflicted with significant error, as the capacitance of the LSC sublayer may change significantly upon deposition of the working electrode.

Table 1: Chemical capacitance values for the thin films deposited during i-PLD measurements.

material	C_{chem} (F/cm^3)
$La_{0.6}Sr_{0.4}CoO_{3-\delta}$	~1700
$La_{0.6}Sr_{0.4}FeO_{3-\delta}$	~1600
$(La_{0.6}Sr_{0.4})_{0.98}Pt_{0.02}FeO_{3-\delta}$	~1600
$SrTi_{0.3}Fe_{0.7}O_{3-\delta}$	~2000
$Pr_{0.1}Ce_{0.9}O_{2-\delta}$	~3000
$La_{0.8}Sr_{0.2}MnO_{3-\delta}$	~65

S.I.2 Effect of Impurity Contaminations on Reaction Rates

To assess the effect of contaminations on the analysis of reaction mechanisms, one LSC thin film was analyzed at $5{\cdot}10^{-6}$ mbar and 600 °C by x-ray photoelectron spectroscopy (XPS) while performing electrochemical impedance spectroscopy. The XPS measurement showed that no contaminations were present on the surface of the thin film and the resistance value lines up perfectly with the slope measured in-situ at low oxygen partial pressures. However, samples measured ex-situ (sulphur contamination on ex-situ samples confirmed previously by XPS [1]) not only exhibit vastly inferior oxygen exchange kinetics but also a different effective $p(O_2)$ dependency [2]. As adsorption kinetics are usually $p(O_2)$ dependent, we suspect that many measurements of the oxygen exchange kinetics are afflicted by significant uncertainty due to contaminations by impurities in the measurement atmosphere.

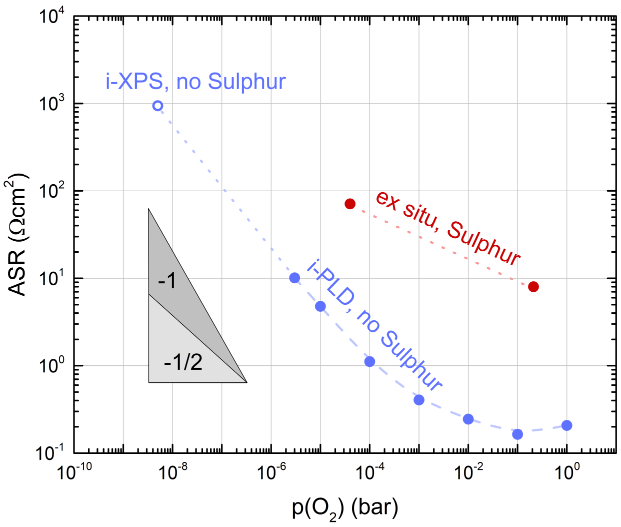

Figure 1: Area specific resistance of LSC during IPLD, at very low $p(O_2)$ during in-situ XPS and measured in a common ex situ setup [2].

S.I.3 In-Situ Degradation Phenomena

In Fig. 2, the $p(O_2)$ dependence of the area specific resistance is shown for measurements from low to high oxygen partial pressures and for measurements in the reverse direction. At high oxygen partial pressures, the resistance slowly increases with time for all materials except PCO, probably due to segregation of Sr from the crystal lattice to the surface. This segregation seems to be partially reversible as the resistance decreases slowly at lower oxygen partial pressures, explaining the slight deviation between the curves.

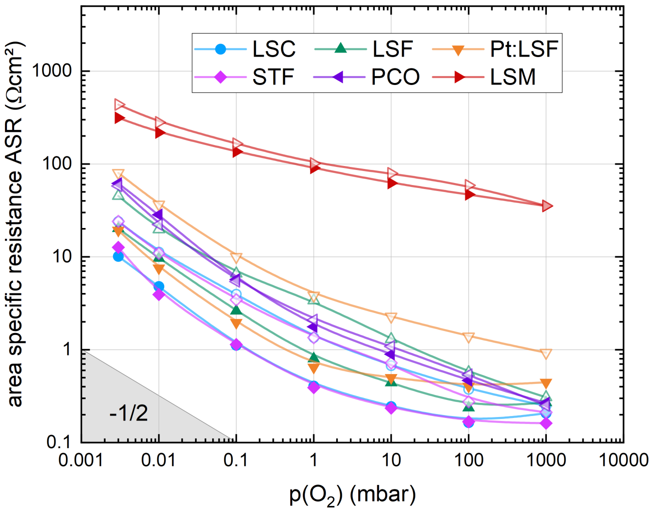

Figure 2: p(O_2) dependence of the surface exchange reaction plotted measured from low to high pressures (full symbols) and in the reverse direction (empty symbols).

S.I.4 Bias impedance measurements

To extract the true potential drop at the working electrode, it is essential that the semicircles corresponding to the surface exchange resistance in parallel to the chemical capacitance of the working electrode thin film and the counter electrode respectively are well separated. At 0.01 mbar, this is only the case for anodic bias voltages and with increasing bias voltage, this separation is becoming even more pronounced. An exemplary impedance curve measured during the application of 200 mV anodic bias is shown in Fig. 3.

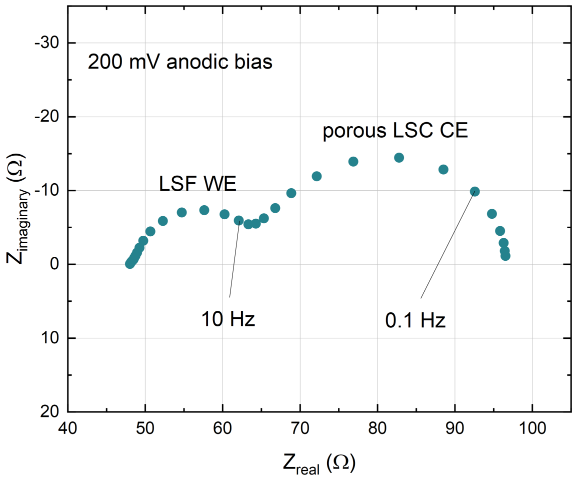

Figure 3: Impedance curve of an LSF thin film under 200 mV anodic bias at 600 °C and 0.04 mbar p(O_2). The two features correspond to the surface exchange resistance in parallel to the chemical capacitance of the LSF thin film and the porous LSC counter electrode respectively.

In Fig. 4, current-overpotential curves recorded during bias impedance measurements are shown. During the measurements, bias was applied until the oxygen vacancy concentration reached a previously defined level (for anodic and cathodic direction respectively), the exact procedure is detailed in the main manuscript.

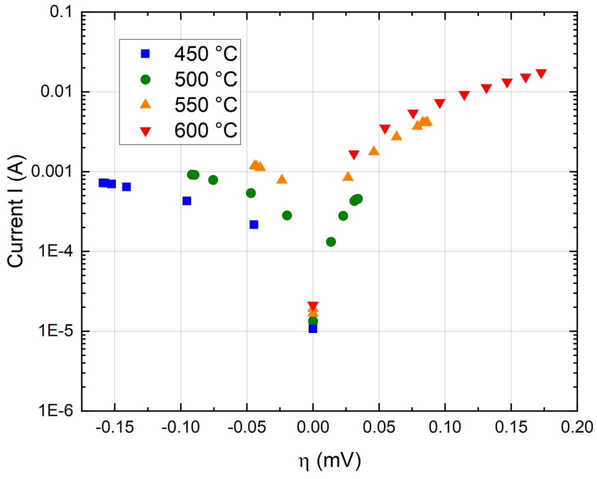

Figure 4: Current-overpotential curves recorded during bias impedance measurements for the evaluation of concentration amended activation energies.

S.I.5 Defect Chemistry of SOFC cathode materials

Defect model of LSF

The most commonly used defect model for bulk LSF was introduced by Mizusaki et al. [3, 4] with oxygen vacancies $V_O^{\cdot\cdot}$ being the main ionic defect together with electrons Fe'_{Fe} and electron holes $\mathrm{Fe}^{\cdot}_{Fe}$ as electronic charge carriers. The equations governing the defect chemistry of LSF are the overall oxygen exchange reaction, the electron-hole pair formation and the according mass action laws as well as charge neutrality and the Fe balance:

$$\frac{1}{2}O_2 + V_O^{\cdot\cdot} \rightleftharpoons O_O^x + 2Fe^{\cdot}_{Fe} \quad \text{with} \quad \frac{[O_O^x][Fe^{\cdot}_{Fe}]}{\sqrt{p(O_2)}[V_O^{\cdot\cdot}][Fe^x_{Fe}]} = e^{\frac{T\Delta S_{ox}-\Delta H_{ox}}{RT}} \tag{1}$$

$$2Fe^x_{Fe} \rightleftharpoons Fe^{\cdot}_{Fe} + Fe'_{Fe} \quad \text{with} \quad \frac{[Fe^{\cdot}_{Fe}][Fe'_{Fe}]}{[Fe^x_{Fe}]^2} = e^{\frac{T\Delta S_i-\Delta H_i}{RT}} \tag{2}$$

$$2[V_O^{\cdot\cdot}] + [h^{\cdot}] = x + [e'], \tag{3}$$

$$[Fe^{\cdot}_{Fe}] + [Fe'_{Fe}] + [Fe^x_{Fe}] = 1, \tag{4}$$

with x being the doping concentration of Sr and the subscript *ox* and *i* denote the reaction enthalpies and entropies of the oxygen exchange reaction and the band-band excitation respectively. Values for the reaction enthalpies and entropies were initially given by Mizusaki et al. [3, 4] and further adapted for thin films by Schmid et al. [5] ($\Delta H_{ox} = -94$ kJmol^{-1}, $\Delta S_{ox} = -50$ Jmol^{-1}K^{-1} and $\Delta H_i = 26$ kJmol^{-1}, $\Delta S_i = -81$ Jmol^{-1}K^{-1}). The calculated charge carrier concentrations for a composition of $La_{0.6}Sr_{0.4}FeO_{3-\delta}$ for [LSF]= $1.66 \cdot 10^{22}$ (calculated for a cubic lattice constant of 3.92 Å[6]) are shown in Fig. 5.

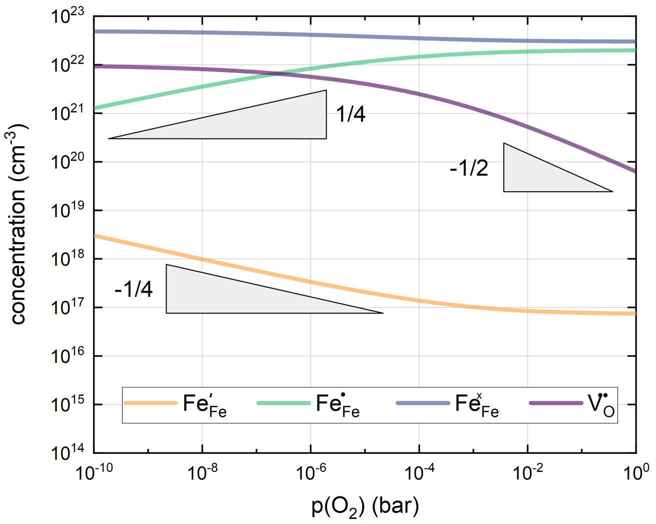

Figure 5: Calculated charge carrier concentrations of $La_{0.6}Sr_{0.4}FeO_{3-\delta}$.

Defect model of PCO

In contrast to LSF and STF, PCO crystallizes in the fluorite structure and is usually considered a solid solution of praseodymium oxide and cerium oxide. Again the oxygen nonstoichiometry is governed by the oxygen exchange reaction which includes charge transfer from Pr atoms. In literature, the electronic structure of PCO is often described by introducing an additional Pr-impurity band which allows the migration of electronic species from one to a neighbouring Pr atom [7]. The defect chemistry of PCO is well explored and the governing equations and the according mass action laws are oxygen exchange, band-band excitation, praseodymium ionization, a simplified charge neutrality equation and mass balances:

$$O_O^x + 2Pr_{Ce}^x \rightleftharpoons \frac{1}{2}O_2 + V_O^{\cdot\cdot} + 2Pr_{Ce}' \quad \text{with} \quad \frac{\sqrt{p(O_2)}[V_O^{\cdot\cdot}][Pr_{Ce}']^2}{[O_O^x][Pr_{Ce}^x]^2} = K_{red,0}e^{-\frac{\Delta H_{red,0}+f\delta}{k_B T}} \tag{5}$$

$$nil \rightleftharpoons h^{\cdot} + e' \quad \text{with} \quad [h^{\cdot}][e'] = K_{i,0}e^{-\frac{E_g}{k_B T}} \tag{6}$$

$$Pr_{Ce}^x + e' \rightleftharpoons Pr_{Ce}' \quad \text{with} \quad \frac{[Pr_{Ce}']}{[Pr_{Ce}^x][e']} = K_{pr,0}e^{-\frac{\Delta H_{Pr}}{k_B t}} \tag{7}$$

$$2[V_O^{\cdot\cdot}] = [Pr_{Ce}'] \tag{8}$$

$$[Pr'_{Ce}] + [Pr^x_{Ce}] = x[PCO] \tag{9}$$

$$[O^x_O] + [V^{\cdot\cdot}_O] = 2[PCO] \tag{10}$$

with x being the dopant concentration of Pr and the subscripts *red* and *pr* denote the reaction enthalpies of the oxygen reduction and the praseodymium ionization respectively. The simplified charge neutrality equation accounts for the fact, that the concentrations of e' and $h^{\cdot}$ are at least two orders of magnitude lower than all other concentrations in the considered p(O_2) range. Mass action constants were again extracted from literature [8, 7] ($K_{red,0} = 0.9 \cdot 10^6$ bar$^{1/2}$, $\Delta H_{red,0} = 1.8$ eV, $f = -4.5$ eV, $K_{i,0} = 6.45 \cdot 10^{44}$ cm^{-6}, $E_g = 3.5$ eV, $K_{pr,0} = 8.18 \cdot 10^{-24}$ cm^3, $\Delta H_{Pr} = -1.43$ eV, $[PCO] = 2.52$ cm^{-3}). The calculated charge carrier concentrations of PCO are depicted in Fig. 6.

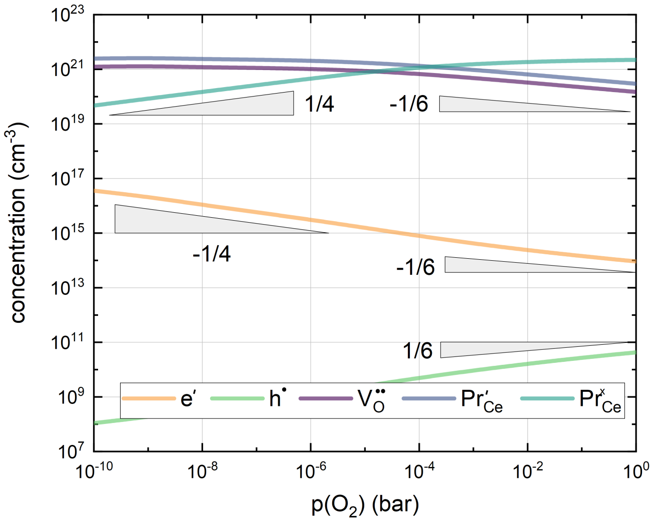

Figure 6: Charge carrier concentrations of $Pr_{0.1}Ce_{0.9}O_{2-\delta}$

S.I.6 p(O_2) dependencies of different reaction mechanisms

To better understand the reaction order with regard to p(O_2), it is necessary to incorporate the complex defect chemistry of the investigated materials into mechanistic considerations. In Fig. 7, a normalized reaction rate is plotted over p(O_2) for different proportionalities to selected defect species of LSF. One sees immediately that different defects partaking in or before the rate limiting step have qualitatively very different impacts on the p(O_2) dependency. For example, when atomic oxygen is partaking in a charge transfer process before or during the rate limiting step, the

slope should not exceed 0.5, in contrast to reactions including molecular oxygen. Moreover, due to the complex defect chemistry, defects affect the p(O_2) dependency differently according to the investigated p(O_2) regime. Nevertheless, the qualitative behaviour of the reaction rate with regard to oxygen partial pressure, when observed over a wide p(O_2) range, gives detailed insight into the actual oxygen exchange reaction mechanism and the partaking defects. It is important to note, that when dealing with equilibrium reaction rates, both directions of the reaction must show the same p(O_2) dependence as the rate limiting step must be the same. Thus, every argument given in this study formulated for the oxygen incorporation reaction translates directly to the reverse direction, i.e. oxygen release, except when discussing non-equilibrium measurements.

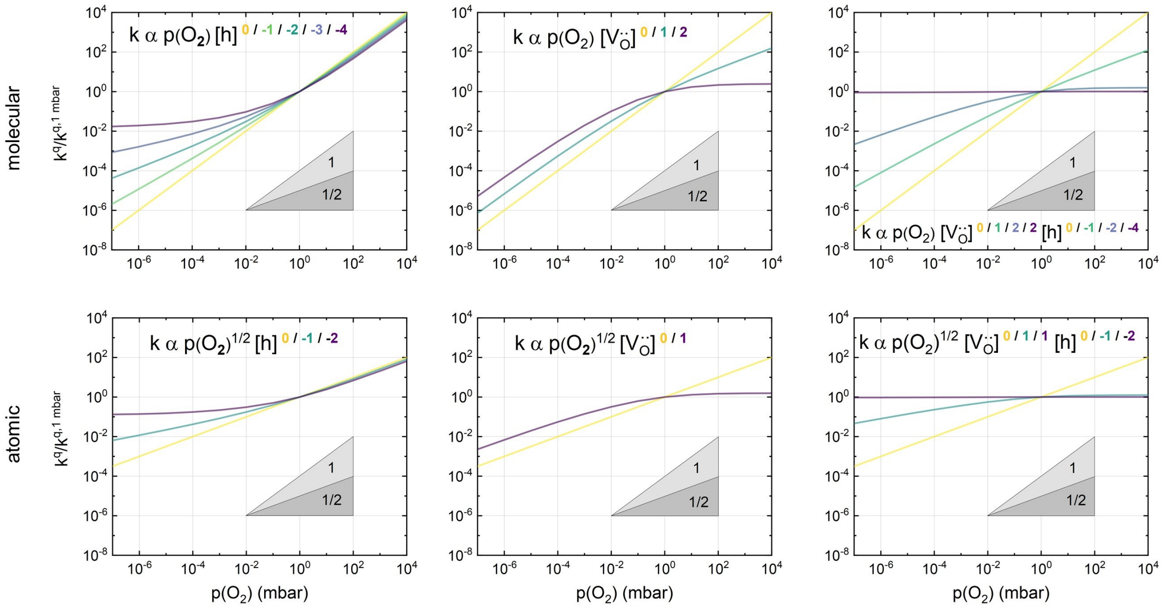

Figure 7: Visualization of the concentration dependency of the oxygen exchange reaction rate for different products of species concentrations in LSF. The colour of the exponent matches the colour of the corresponding line.

S.I.7 Concentration dependent contributions to the effective activation energy

The effectively measured activation energy during equilibrium experiments is naturally a convolute of an actual activation barrier and concentration dependent contributions, originating from the defect chemistry of the material. Thus, activation energies derived from equilibrium measurements are generally not suitable to discuss the actual energetic activation of the oxygen exchange reaction and the defect chemical contributions must be considered in such discussions. For illustrative pur-

poses, these defect chemical contributions are shown for the two exemplary rate determining steps of the main paper. Here, it is important to distinguish between incorporation and release reaction, as different species partake in the reaction.

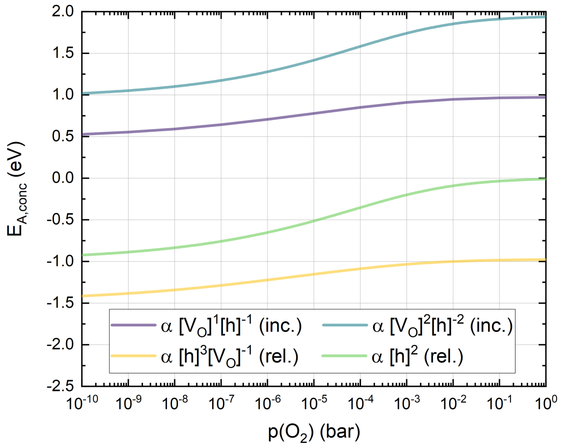

Figure 8: Concentration dependent contributions to the effective activation energy shown for the incorporation and the release direction of two different rate determining steps and the corresponding proportionalities to defect concentrations.

References

[1] Alexander Schmid, Andreas Nenning, Alexander Opitz, Markus Kubicek, and Jürgen Fleig. "High Oxygen Exchange Activity of Pristine $La_{0.6}Sr_{0.4}FeO_{3-\delta}$ Films and Its Degradation". In: *Journal of The Electrochemical Society* 167.12 (2020), p. 124509.

[2] Matthäus Siebenhofer, Tobias Martin Huber, Gernot Friedbacher, Werner Artner, Jürgen Fleig, and Markus Kubicek. "Oxygen exchange kinetics and nonstoichiometry of pristine $La_{0.6}Sr_{0.4}CoO_{3-\delta}$ thin films unaltered by degradation". In: *Journal of Materials Chemistry A* 8.16 (2020), pp. 7968–7979.

[3] Junichiro Mizusaki, Masafumi Yoshihiro, Shigeru Yamauchi, and Kazuo Fueki. "Nonstoichiometry and defect structure of the perovskite-type oxides $La_{1-x}Sr_xFeO_{3-\delta}$". In: *Journal of Solid State Chemistry* 58.2 (1985), pp. 257–266.

[4] Junichiro Mizusaki, Masafumi Yoshihiro, Shigeru Yamauchi, and Kazuo Fueki. "Thermodynamic quantities and defect equilibrium in the perovskite-type oxide solid solution $La_{1-x}Sr_xFeO_{3-\delta}$". In: *Journal of Solid State Chemistry* 67.1 (1987), pp. 1–8.

[5] Alexander Schmid, Ghislain M Rupp, and Jürgen Fleig. "Voltage and partial pressure dependent defect chemistry in (La, Sr) $FeO_{3-\delta}$ thin films investigated by chemical capacitance measurements". In: *Physical Chemistry Chemical Physics* 20.17 (2018), pp. 12016–12026.

[6] Thomas Götsch, Lukas Schlicker, Maged F Bekheet, Andrew Doran, Matthias Grünbacher, Corsin Praty, Mizuki Tada, Hirosuke Matsui, Nozomu Ishiguro, Aleksander Gurlo, et al. "Structural investigations of $La_{0.6}Sr_{0.4}FeO_{3-\delta}$ under reducing conditions: kinetic and thermodynamic limitations for phase transformations and iron exsolution phenomena". In: *RSC Advances* 8.6 (2018), pp. 3120–3131.

[7] Sean R Bishop, Todd S Stefanik, and Harry L Tuller. "Electrical conductivity and defect equilibria of $Pr_{0.1}Ce_{0.9}O_{2-\delta}$". In: *Physical Chemistry Chemical Physics* 13.21 (2011), pp. 10165–10173.

[8] Di Chen and Harry L Tuller. "Voltage-Controlled Nonstoichiometry in Oxide Thin Films: $Pr_{0.1}Ce_{0.9}O_{2-\delta}$ Case Study". In: *Advanced Functional Materials* 24.48 (2014), pp. 7638–7644.
